# A network approach for low dimensional signatures from high throughput data

**DOI:** 10.1038/s41598-022-25549-9

**Published:** 2022-12-23

**Authors:** Nico Curti, Giuseppe Levi, Enrico Giampieri, Gastone Castellani, Daniel Remondini

**Affiliations:** 1 Department of Physics and Astronomy, University of Bologna, Bologna, Italy; 2 INFN Bologna, Bologna, Italy; 3 Department of Experimental, Diagnostic and Specialty Medicine, University of Bologna, Bologna, Italy

**Keywords:** Data processing, Software, Statistical methods, Complexity, Software, Complex networks, Biological physics

## Abstract

One of the main objectives of high-throughput genomics studies is to obtain a low-dimensional set of observables—a signature—for sample classification purposes (diagnosis, prognosis, stratification). Biological data, such as gene or protein expression, are commonly characterized by an up/down regulation behavior, for which discriminant-based methods could perform with high accuracy and easy interpretability. To obtain the most out of these methods features selection is even more critical, but it is known to be a NP-hard problem, and thus most feature selection approaches focuses on one feature at the time (k-best, Sequential Feature Selection, recursive feature elimination). We propose DNetPRO, *Discriminant Analysis with Network PROcessing*, a supervised network-based signature identification method. This method implements a network-based heuristic to generate one or more signatures out of the best performing feature pairs. The algorithm is easily scalable, allowing efficient computing for high number of observables ($10^3$–$10^5$). We show applications on real high-throughput genomic datasets in which our method outperforms existing results, or is compatible with them but with a smaller number of selected features. Moreover, the geometrical simplicity of the resulting class-separation surfaces allows a clearer interpretation of the obtained signatures in comparison to nonlinear classification models.

## Introduction

The huge dimensionality of omics data (e.g. microarray or NGS transcriptomics, epigenomics, SNP profiling, proteomics, metabolomics, metagenomics of gut microbiota) poses enormous challenges as how to extract useful information from them. One of the prominent problems is the identification of a “signature”, i.e., a low-dimensional set of features (such as the measured biological probes) for classification and diagnostic purposes. This can be used for example to better stratify patients for personalized intervention strategies based on their molecular profile^1–4^.

**Figure 1:**
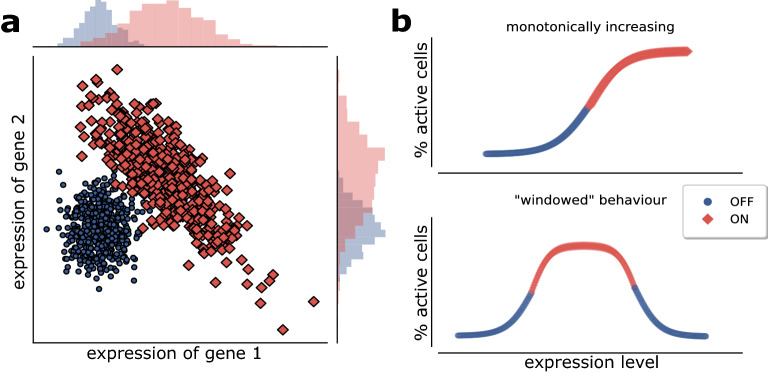
(**a**) A simple 2D ideal model in which single-feature classification performance fails in predicting higher-dimension classification performance. Both features (*gene expression 1* and *gene expression 2*) badly classify in 1D but have a very good performance in 2D. Moreover, the classification can be easily interpreted in terms of combined higher/lower expression of both probes. (**b**) Activity of a biological feature (e.g. a gene) as a function of its expression level: top—monotonically increasing, often also dichotomized to an on/off state; bottom—“windowed” behavior, in which the two activity states do not depend monotonically on expression levels. X axis: expression level, Y axis: biological state (arbitrary scales).

Many approaches are used for these classification purposes^5^, such as Support Vector Machine, K-nearest Neighbor, Neural networks, Penalized regression (ridge, LASSO and Elastic Net^6^) and Random Forest^7^. These methods typically under-perform when a high number of features is provided (due to problem under-specification and curse of dimensionality), such as in the high-throughput biological data, and therefore need to be preceded by some form of feature engineering method. Some methods build signatures by means of single-feature scoring methods^8,9^ (e.g. inferential testing for two-class comparison) but these approaches could fail even in simple 2-dimensional situations. An example is shown in Fig. 1a, in which both features perform poorly when taken individually, but their performance becomes optimal in a 2-dimensional combination, through a simple linear separation of the two classes. Others methods search for projections or nonlinear feature combinations in a latent space, but these approaches can reduce the explainability of the results, and typically require a large amount (if not all) of the original features to be included.

It is known that complex separation surfaces characterize classification tasks associated to image and speech recognition, for which Deep Networks have been successfully applied in recent times^10^. On the contrary many biological data, such as gene or protein expression, are more likely characterized by an up/down regulation behavior (as shown in Fig. 1b top), while more complex patterns (e.g. a “windowed” optimal range of activity, Fig. 1b bottom) are much less common^11^. Thus, discriminant-based methods (and logistic regression methods alike) could provide good classification performances in these cases if applied in at least 2-dimensional spaces to account for situations as shown in Fig. 1a. Moreover, the “linearity” of the proposed methods (that generate very simple class separation surfaces, i.e., linear or quadratic) guarantees that the construction of a multidimensional signature based on feature pairs is feasible and amenable of a simple explanation in terms of combined feature up/down regulation.

A possible way to overcome these issues was introduced by Geman et al.^12^ via the Top Scoring Pair (TSP) classifier and its further refinements^13,14^ and extensions^15^. The TSP algorithm is based on a bottom-up combinatorial approach that exploits the discriminant power of all feature pairs tailored for gene expression classification problems: TSP algorithm identifies pairs of features whose relative expressions/values are upturned between two classes, i.e., it tries to find couples of genes whose relative rankings are inverted in most samples of the two classes. The simplicity of the method guarantees an easy interpretation of the results, but it does not provide any criteria to combine several gene-pairs into a higher-dimensional signature.

**Figure 2:**
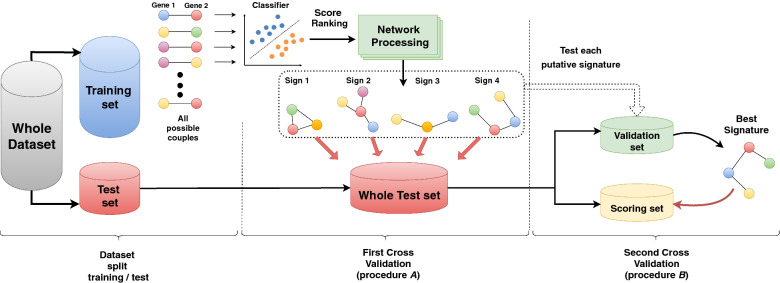
Scheme of the DNetPRO algorithm. On the “Training set”, all possible pairs of features are used for Discriminant Analysis, generating a fully connected network with links weighted by pair classification performances. By thresholding the weighted links (i.e., setting to zero links with performance below a defined threshold) one or more signatures are identified as the resulting network connected components. In procedure *A*, signatures’ performance ars evaluated on the “Whole Test set”. In procedure *B*, a unique best signature is identified on a “Validation set” and then tested in a “Scoring set”, obtained by further splitting the “Whole Test set”.

DNetPRO—*Discriminant Analysis with Network PROcessing*—generates multivariate signatures starting from all the feature pairs, tested via Discriminant Analysis (ref. Fig. 2 and Supplementary Material for detailed method description) considering a different signature generation than the TSP algorithm: each couple of omics features (e.g. gene, miRNA protein expression levels, etc.) constitutes two nodes of a network, and a link between them is created if their classification performance exceeds a selected threshold (see “Methods” section). Given this set of links, many connected subnetworks can be generated. The nodes (features) of each one of these subnetworks constitute one of the putative classification signatures. Extensive exploration of all possible feature combinations (all *K*-tuples over *N* possible features) is known to be an NP-hard problem^16^; the DNetPRO method is an attempt to overcome single feature selection without the computational burden of the full combinatorial exploration, with a computing time for feature space exploration proportional to the square of the number of features (ranging from $10^3$ to $10^5$ in a typical high-throughput omics study). Moreover, the geometrical simplicity of the resulting class-separation surfaces allows an easier interpretation of the results compared to very powerful methods like nonlinear-kernel SVM or Neural Networks that suffer from hard-to-explain decision boundaries. The linearity of the separation surface means that the resulting signature generates a single score obtained by the linear combination of the used features, with a single threshold value to separate the two classes. DNetPRO method belongs to the category of network-based algorithms, a class of methods recently applied for dimensionality reduction, visualization and clustering tasks that exploits the heuristics provided by a network representation of available data^17–19^.

## Results

We tested the proposed DNetPRO algorithm on synthetic data and on public cancer omics datasets of different types (Synapse datasets: mRNA, miRNA, and RPPA) in comparison with a set of current state-of-art classifiers applied on them in a recent paper^20^. To compare our results with theirs, we used the AUC (*Area Under the Curve*) score, provided in the paper as the result of their analyses. All datasets were analyzed considering the pipeline proposed in Fig. 2, that tried to follow as closely as possible the methods described in the paper.

### Synthetic data

We compare the accuracy performance of a classical incremental feature selection algorithm, i.e., single feature selection based on ANOVA, with the proposed DNetPRO algorithm, on a synthetic toy model dataset (see “Methods” section for its description). For both the features selection methods we used a diag-quadratic Discriminant Analysis for the evaluation of classification performances. The simulations were performed according to the procedure *A* showed in Fig. 2.

**Figure 3:**
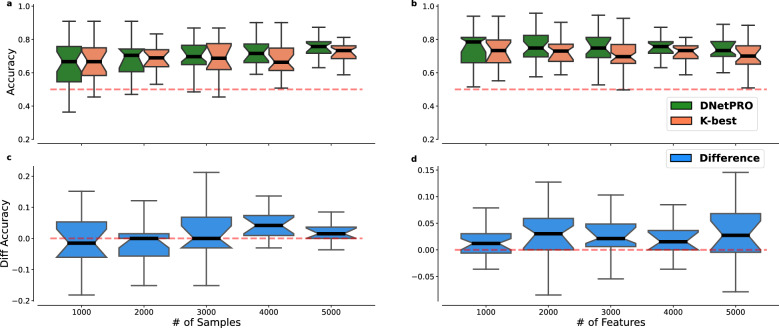
Synthetic dataset simulation. Comparison of accuracy performances obtained by the DNetPRO algorithm (green) and the *K*-best algorithm (red). (**a**) Performances obtained as a function of the number of samples, keeping fixed the number of features (4000 features). (**b**) Performances obtained in function of the number of features, keeping fixed the number of samples (500 samples). (**c,d**) Differences between the performances obtained by the DNetPRO and the *K*-best algorithm on the same simulations.

For the same number of features (Fig. 3a,c) two methods perform quite similarly, but the DNetPRO obtains better performances as the number of samples increases.

When the number of features is varied while keeping fixed the number of samples (Fig. 3b), DNetPRO always outperforms the *K*-best algorithm in terms of median accuracy (black line in the plot, ref. Fig. 3d). As the number of features increases, the efficiency of the DNetPRO algorithm also increases, until it exceeds the *K*-best algorithm (and its distribution is narrowed). We reached this situation quite rapidly in our simulations, since we constrained our toy model with a forced unbalance between the number of samples and features, i.e., the so-called ill-posed problems. DnetPRO thus allows to identify couples of features with good performance that rank low singularly, highlighting their synergistic behaviour.

### Synapse dataset

Four omics dataset were extracted from TCGA (The Cancer Genome Atlas) and made available at the Synapse homepage created by Yuan et al.^20^ (syn300013, doi:10.7303/syn300013): kidney renal clear cell carcinoma (KIRC), glioblastoma multiforme (GBM), ovarian serous cystadenocarcinoma (OV) and lung squamous cell carcinoma (LUSC) (see “Materials and methods” and Supplementary Materials for further details).

**Figure 4:**
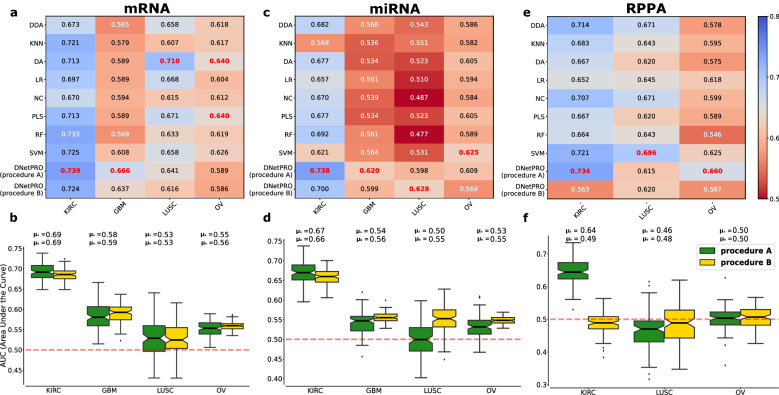
Results obtained by the DNetPRO on the mRNA, miRNA, and RPPA samples related to the four cancer types in the Synapse dataset. Methods legend: *DDA* diagonal discriminant analysis, *KNN* K-nearest neighbor, *DA* discriminant analysis, *LR* logistic regression, *NC* nearest centroid, *PLS* partial least squares, *RF* random forest, *SVM* support vector machine. (**a,c,e**) Comparison of the DNetPRO results with the methods used in the work of Yuan et al., in terms of the maximum AUC value obtained on a ten-fold cross-validation procedure (bold red: top-performing method). In procedure *A*, the maximum AUC was estimated as the maximum score value obtained by each extracted signature on the related test set of the ten-fold subdivision. In procedure *B*, the maximum AUC was estimated as the maximum score value obtained by the best signature obtained by the ten-fold cross-validation on the related validation set. (**b,d,f**) Distributions of the AUC values related to each analyzed dataset. Green boxplots: results using procedure *A* as described in Fig. 2; yellow boxplots: results using procedure *B*. The results obtained by the two procedures are not directly comparable due to the different data subdivisions and therefore the two distributions are plotted individually.

For each cancer type, we analyzed the mRNA, miRNA, and RPPA (protein) datasets, performing the classification of dichotomized survival outcomes^20^ via the same train/test subdivisions provided by Yuan et al. We intentionally did not include the set of clinical information related to each cancer type (and available in the online repository), since the focus of our work is not on the best classification performances for the involved tumor, but only on the possibility of using this approach to omics feature selection on high throughput data. As already showed in the work of Yuan et al., we could surmise that extending the analysis to other types of information (clinical, demographical or other) could improve the overall classification performance. We report the results in terms of maximum AUC (ref. Fig. 4a,c,e) and AUC distributions (ref. Fig. 4b,d,f) in order to be comparable with the results reported by the algorithms used in the work of Yuan et al. We show the results obtained with the procedure *A* of DNetPRO (corresponding to the validation approach used by Yuan et al.) and procedure *B* (full double cross-validation procedure to avoid data contamination) in comparison with a series of more complex methods based on single-feature evaluations as proposed by Yuan et al.

The results on the mRNA datasets using procedure *A* are comparable (LUSC) or better (KIRC, GBM) than the results reported in Ref.^20^, except for the OV dataset. This ranking is maintained even with the more conservative procedure *B*, involving a further cross-validation step. The size of the extracted signatures is approximately constant between cross-validations, and typically smaller than 500 genes in each pipeline execution. Performances decrease with the introduction of the second cross validation step (procedure *B*), as expected, but they remain quite stable, showing the robustness of the extracted signatures. We remark that the validation procedure used by Yuan et al. corresponds to our approach without the second validation step (procedure *A*). The results obtained on the miRNA datasets are comparable to the reference ones, while for the RPPA datasets only the LUSC shows AUC values comparable with the others. Moving from procedure *A* to procedure *B*, i.e., adding a second cross-validation step, RPPA performances drastically decrease for the KIRC and OV, while they remain stable for the LUSC dataset. The same behavior is shown in the miRNA datasets, in which however both performances are still comparable or better (KIRC, GBM, LUSC) than the reference ones.

### Signature overlap

To test the stability of the identified signature, we repeated 100 times a cross-validation procedure, extracting a set of 1000 independent signatures (see Fig. 2). We focused on the KIRC mRNA dataset, in which the extracted signatures ranged from 4 to 654 genes (and an average of $\mu =382$ genes), and we counted the occurrence of each gene among the 1000 signatures. The same analysis was performed considering the signatures generated using the *K*-best score features based on ANOVA, and a random feature extraction as a null model. Both DNetPRO and *K*-best identified a core set of genes common to all the signatures, significantly differing from the null model.

We observed a set of 74 genes in 90% of DNetPRO signature (20 of which are common to all signatures). This list of 74 genes was mapped into the TISIDB^21^ on-line database, confirming the relationship between most of them (65/74, i.e., the 87% of the genes identified by the DNetPRO algorithm) and KIRC tumor (see Supplementary Material for further details). Thus we verified that the results of the signature identification procedure provided by DNetPRO are quite stable, and the genes found are also biologically relevant for the studied case.

## Discussion

In this work we proposed a network-based combinatorial feature extraction method, the DNetPRO algorithm, that combines top ranking pairs of omics entities (e.g. gene transcripts or proteins) into a multidimensional classification signature. This methods appears particularly fit to omics data (at difference with more complex data types such as text or images) in the hypothesis of monotonic changes in feature values between dichotomics classes, that allows a simple biological interpretation of the resulting signature in terms of up/down regulation of its elements. We tested our method on synthetic data, showing how its efficiency increases on ill-posed problems (similar to those encountered in omics analysis) in comparison with classical incremental feature selection approaches. Moreover, the proposed DNetPRO method was also tested on benchmark real datasets, with results in general better or comparable with many state-of-art classification methods.

From these analyses it can be seen that, given a similar (or better) classification performance, DNetPRO allows a simple biological interpretation of the identified signatures, in terms of up/down regulation of its elements. Thus DNetPRO uses a network-based approach to merge the top-scoring feature pairs into a unique multivariate signature, in alternative to the TSP algorithm that considers disjoint top-ranking couples without any overlap. In our case, given the linearity of the class separating surface (as provided by Discriminant Analysis) we can provide a joint interpretation of up or down regulation for all signature’s features, at difference with TSP for which this interpretation can be given only for all feature couples separately, with the possibility to identify larger modules of features involved in the studied mechanism.

At difference with the TSP approach, that identifies disjoint couples of omics features as a signature, one possible limit of the proposed method is that multiple highly correlated features could be included in the same signature. Albeit this behavior means that redundant information could be present in the identified signatures, this does not necessarily represent a major disadvantage, because it could add robustness to the signature when experimental noise (or individual variability) affects part of the selected features. Moreover, once the whole multidimensional signature is obtained by the network approach, signature performance could be tested while removing some of the features, picking them randomly one by one or in groups, or following a network approach. For example, the signature seen as a network could be “pruned” by removing pendant nodes (i.e., features connected to the signature by just one link) and this procedure could be repeated iteratively until reaching the network “core” (i.e., all nodes with a degree $\ge 2$). The heuristic approach to reduce signature size based on network node properties could be also applied by keeping only features with highest node centrality, such as degree or betweenness centrality, but its validity in real cases needs to be tested, in the hypothesis that the more a feature is central in the signature network, the more it is relevant to classification performance.

We remark that the signatures identified with DnetPRO have a purely statistical relevance, being generated with a purpose of maximal classification performance, but previous applications of a simplified version of the DNetPRO algorithm^1,22–24^ allowed to gain useful knowledge on the underlying biological mechanisms, in the hypothesis that omics features with largest changes between classes (thus with highest discriminatory power) are more strongly associated to the studied phenomena.

Finally, from a computational point of view we remark that the method is easily scalable on parallel architectures, since the feature couple testing can be performed separately on different cores, allowing fast processing of high-dimensional data (in the order of $10^4$ elements in 1 min on server grade machines, see Supplementary Material) and a version of the algorithm is publicly available on Github^25^.

## Methods

### DNetPRO algorithm

The pseudo-code of the proposed DNetPRO algorithm could be sketched as:
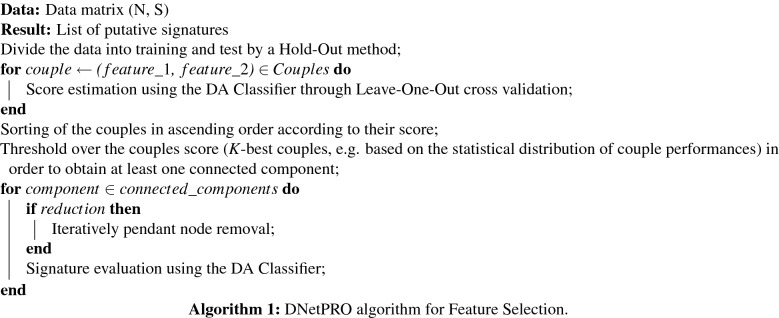


Given a *dataset*, consisting of *S* *samples* (e.g., cells, patients) with *N* observations each (our *features*, e.g., omics measurements such as gene, protein or metabolite expression) the signature identification procedure is summarized with the following pipeline:

- Separation of available data into a *training* and a *test* set (typically 66/33, or 80/20).
- Estimation of the classification performance according to the desired metric on the training set of all $S(S-1)/2$ *feature pairs* through a computationally fast and reproducible cross-validation procedure (leave-one-out cross validation was chosen). In this work we used the Matthew Coefficient as a metric for performance estimation of a discriminant analysis. The results are mapped into a fully connected symmetric weighted network, with nodes corresponding to features and link weights corresponding to performance of the node couples.
- Selection of classification signature(s) through a hard-thresholding procedure, that removes links (and nodes) from the initial fully connected network: every *connected component* obtained is considered as a putative classification signature. The threshold value can be tuned according to a desired minimum-performance value or considering a minimum number of nodes/features in the signature. The threshold value can be determined also by testing each of the obtained performances as a possible cut-off via a cross validation of the entire signature extraction procedure.
- (Optional) In the hypothesis that node connectivity is associated to the global feature performance in combination with the other features, to reduce the size of an identified signature, the *pendant nodes* of the signature network, i.e., nodes with degree equal to one, can be removed. This procedure can be applied once, or recursively until the core network, i.e., a network with all nodes with at least two links, is reached. We have tested the efficacy of this empirical approach in some real cases^11,26^, obtaining a smaller-dimensional signature with comparable performance, even if we remark that this is an empirical result lacking a solid theoretical basis and further tools could be used for feature selection like the SHAP algorithm^27^.
- (Optional) The classifier used in the feature selection and the final classification does not need to be a Discriminant Analysis classifier, but can in principle be any classifier. Moreover, the classifier used in the feature selection does not need to be the same one used for the final evaluation of the obtained signature. We did not test this option but maintained the same method in all the algorithm steps where it was needed.
- All signatures are validated onto the test set, obtaining more than one final signature with its own estimated performance, and this corresponds to procedure *A* in Fig. 2. Eventually, these signatures can be further characterized, for example by their biological significance, or they could be considered as different “disease modules”^28,29^.
- To identify a unique final signature (procedure *B* in Fig. 2) after training, the dataset can be further split into a test set (to identify the best signature) and validation set (to evaluate its performance).

To test the performance of all feature pairs, we used a diag-quadratic Discriminant Analysis, a robust classifier that allows fast computation.

A variant of DNetPRO method has been also applied for dimensional reduction of network structures, where sub-modules of the network were identified by studying the correlation between links^30,31^.

Further information about the implementation of the algorithm are available in the Supplementary Material.

DNetPRO code is publicly available on Github^25^ as C++ library and Python module.

### Synthetic data

The most common feature selection algorithms usually treat features as individual and independent entities. Starting from the features ranked according to their scores, a signature is obtained selecting the top ones according to an incremental addition of features until a desired output performance is reached. These methods are called *K*-best algorithms, and they select the features without any information on their mutual interaction or correlation. The proposed DNetPRO algorithm tries instead to extract the more statistically significant features considering the interaction between them, i.e., their combination in pairs for a 2-dimensional discriminant supervised classification.

To compare the two methods on a synthetic dataset, we used a toy model generator provided by the scikit-learn package, generating normally-distributed clusters of points and introducing interdependence between the features. The model generator creates clusters of points normally-distributed about vertices of a pre-determined number of *informative dimensional* hypercube, and assigns an equal number of clusters to each class (2 in our case). The model generator allows to set the number of sample classes, distinguishing between *informative features*, i.e., features which easily separate the class populations, and *non-informative features*, i.e., features which represent noise in our problem. The number of informative features should be realistically small compared to the noise, so in our simulations we chose to introduce a maximum of 1% informative features in each simulation.

We randomly generated data from Gaussian uncorrelated distributions with an increasing number of samples and features, i.e., dimensions. We want to remark that this configuration of data would tend to prefer the single feature methods like the *K*-best one: the inclusion of noise sources and a high number of dimensions would stress the *K*-best efficiency, representing a good benchmark for the DNetPRO application. In each simulation we split the number of samples in training and test sets (Hold-Out method, with 66% of data as training and 33% as test) and we applied the DNetPRO algorithm. From each simulation we tested the extracted signatures on the test set, keeping the best performing one. On the same data subdivision we applied the *K*-best algorithm, filtering the same number of features of the DNetPRO best signature, i.e., *K* equal to the number of nodes in the DNetPRO best signature. In this way, we can compare the performances obtained on the test set by the two methods, using the same number of features/dimensions for both the algorithms. We used as threshold criteria for the DNetPRO algorithm a maximum number of features: keeping the top scoring pairs, we progressively added groups of features until a maximum number of 100 pairs was reached (ref. Supplementary Materials for a description of ranked distributions). We intentionally did not tune the threshold parameter of the DNetPRO algorithm to keep the comparison of the two methods unbiased.

### Synapse dataset

We processed each cancer dataset by adding the absolute value of a zero-mean Gaussian random noise ($\sigma = 10^{-4}$) to remove possible zero values and to improve the numerical stability of the Discrimant Analysis employed in this work. This procedure does not affect the interpretation of the results, and the applicability of the proposed method to sparse datasets. Then, we randomly split each dataset in training and test sets with a stratified (i.e., balanced for class sample ratio) ten-fold procedure: with this stratification each training set is representative of the whole dataset. The choice of a ten-fold splitting is aimed to reproduce the analysis pipeline presented by Yuan et al.^20^ with an analogous cross-validation procedure. Since we do not have exact details of their data splitting, the cross-validation was repeated 100 times, for a total of 1000 training procedures for each tumor (OV, LUSC, KIRC, GB) and data type (mRNA, miRNA, RPPA). Each training procedure led to the extraction of multiple signatures.

We chose threshold values to obtain a resulting number of features in the signatures in the order of $10^2$–$10^3$. According to each dataset, an appropriate threshold was estimated to achieve this requirement. If more than one connected component existed, each one was considered as a different signature.

The final multidimensional signatures were tested by Discriminant Analysis with a diag-quadratic metrics, to avoid possible problems deriving from covariance matrix non-invertibility (as for the Mahalanobis distance in which there is a higher number of coefficients to be estimated from the data).

### Cross validation procedures A and B

To allow a fair comparison with the results presented by Yuan et al.^20^ we performed an identical cross-validation procedure, referred to as procedure A. This procedure however does not allow an unbiased comparison of the signature performance, lacking a second cross-validation step. To obtain such an unbiased performance estimation we also proceded to evaluate the results using a double cross-validation approach, referred to as procedure B.

In the single cross-validation pipeline (procedure *A*, ref. Fig. 2) the best signature was extracted as the one reaching the highest accuracy score during the training step. This best signature was then tested over the available test set. The introduction of the second cross-validation step (procedure *B* in Fig. 2) led to choose the best signature as the one with the best performances over a subset of the whole test set (renamed *test set*) evaluating the final performance on the remaining *validation set*.

### Signature overlap

The DNetPRO algorithm can provide more than one signature as outcome, given by the various connected components found in the feature network, and a unique top-performing signature can be obtained by a further cross-validation step (procedure *A* and *B* in Fig. 2, respectively).

In our applications, we divided the datasets into a training-test subdivision and the signatures were extracted along a ten-fold cross-validation over the training sets. This kind of setup could, in the worst case, extract up to 10 totally different signatures (one for each split).

Starting from this large number of signatures, we evaluated the robustness of the DNetPRO algorithm in the feature identification, studying the overlap between them. From a statistical point of view, it is quite unlikely that the same set of features would be included into all the extracted signatures, especially on this application in which features represent gene expressions. On the other hand, the overlap of these signatures could highlight a statistical significance of some features, and thus genes related to the studied cancer dataset.

For each fold we evaluated the average dimension of the signatures, and we computed the distribution of these dimensions for each cancer type (see Supplementary Material for further details).

## Supplementary Information

Supplementary Information.

## Data Availability

The synthetic datasets used for the analyses are available from the corresponding author on reasonable request. The Synapse dataset used for the analyses is available at the Synapse homepage (accession number syn300013, doi:10.7303/syn300013). The code for the reproducibility of the results is available on Github^25^.
