# Supplementary material for: A network approach for low dimensional signatures from high throughput data
Source: Sci Rep. 2022 Dec 23;12:22253. doi: 10.1038/s41598-022-25549-9 (PMC9789141; doi:10.1038/s41598-022-25549-9)
Supplement: Supplementary file 1 — Supplementary Information. [file 41598_2022_25549_MOESM1_ESM.pdf]

A network approach for low-dimensional signatures from high-throughput data

Nico Curti[a, 1], **Giuseppe Levi**[b, c, 1], **Enrico Giampieri**[a, 2], **Gastone Castellani**[a], **and Daniel Remondini**[b, c]

[a]Department of Experimental, Diagnostic and Specialty Medicine, University of Bologna
[b]Department of Physics and Astronomy, University of Bologna
[c]INFN Bologna

ABSTRACT

One of the main objectives of many high-throughput studies is to obtain a relatively low-dimensional set of observables - a signature - for sample classification purposes (diagnosis, prognosis, stratification). We propose DNetPRO, *Discriminant Analysis with Network PROcessing*, a supervised network-based signature identification method as extension of the classical kTSP, *k-Top Scoring Pairs*, algorithm for the gene expression classification. The algorithm is easily scalable, allowing efficient computing for high number of observables (10^3–10^5). We show applications on real high-throughput genomic datasets in which our method outperforms existing results or compares to them. but with a smaller number of selected features. Moreover, the geometrical simplicity of the resulting class-separation surfaces allows a clearer interpretation of the obtained signatures in comparison to nonlinear classification models.

DNetPRO algorithm implementation

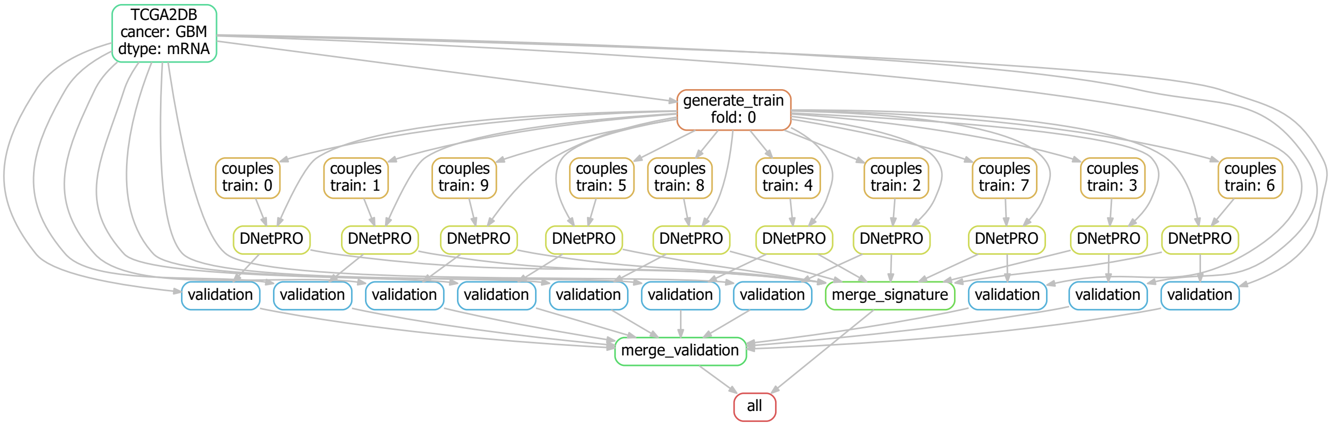

Supp. Fig. 1. Example of DNetPRO pipeline with a single cross validation step. This highlights the independence of each fold. This scheme shows a possible distribution of the jobs on a multi-threading architecture or for a distributed computing architecture. The second case allows further parallelization scheme (hidden in the graph) for each internal step (e.g. the evaluation of each pair of genes).

The DNetPRO algorithm is defined by a series of independent computational processes given by the variable pairs evaluation. This characteristic guarantees an easy parallelization (embarassingly parallel) of the process to increase its speed performances. Therefore, we developed a multi-core parallelized version of the algorithm, with shared memory architecture, splitting the analysis across several threads.

To improve the scalability of our algorithm, we implemented the pipeline scheme using Snakemake[1] rules (Python codes) and the computation of discriminant networks (the most time expensive step) was performed in C++ with the OpenMP multi-threading support. The computational scheme of the proposed pipeline for a single cross-validation step is shown in supp. Fig. 1.

Using Snakemake we can easily distribute our pipeline on several machines via a master-slave parallelization scheme. In

this case each step of supp. Fig. 1 can be performed by a different computer unit, preserving the multi-threading steps, with a maximum scalability and the possibility to enlarge the problem size and the number of variables.

We tested our implementation on a dedicated HPC server (128 GB RAM memory and 2 CPU E5-2620, with 8 cores each), comparing its scalability to a common laptop (8 GB RAM memory and 1 CPU i7-6500U, with 2 cores), using the same dataset (20 530 probes and 150 samples, with a total of more than 2×10^8 combination of variables). Both the tests were performed with the hyper-threading enabled and considering the time performances for the full combination analysis and pairs reordering, i.e., step 1 and 2 of the DNetPRO algorithm. The pairs evaluation took around 1 minute on the server machine and 10 minutes on the laptop, showing a good scalability of the code on the available threads. We want to remark that the loading of the data (included in the time evaluation) was always performed in single thread. Moreover, the sorting algorithms are drastically affected by the CPU cache size, giving a huge advantage to the server machine for this kind of analysis.

We implemented also an MPI (*Message Passing Interface*) version of the algorithm to face analyses of larger datasets, with a greater number of variables. We did not need to use a such parallelization scheme in our simulations, since the multi-threading version on a bioinformatics server was fast enough for our applications.

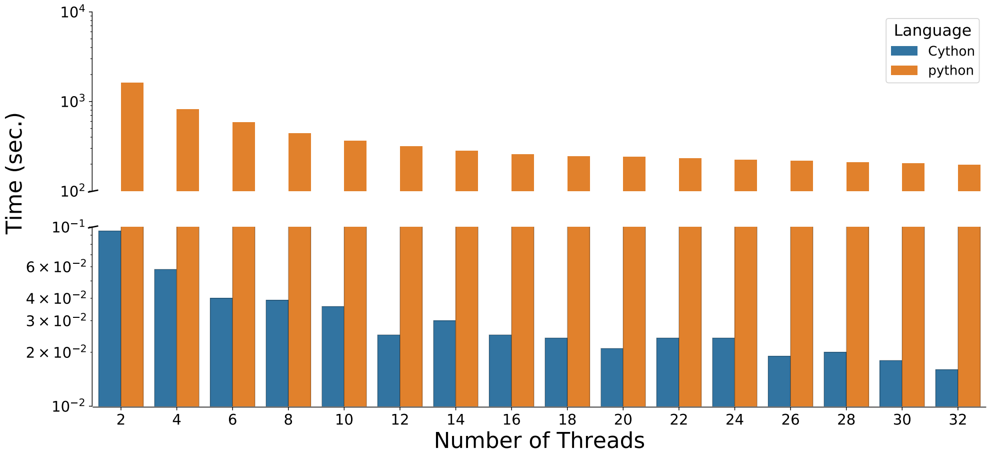

Supp. Fig. 2. Scalability of DNetPRO algorithm implementation across various number of threads, using a dataset composed by 90 samples and 90 variables. We compare the computational time performances of our Cython version of the DNetPRO algorithm against a parallel pure-Python one (with the support of *Numpy* and *Scipy* modules). The oscillatory trend of the Cython times is due to an unbalanced partition of the tasks related to the number of samples provided.

The developed combinatorial algorithm has been also wrapped in Python via Cython[2] to allow a good integration with other common machine learning tools and the most used Python libraries such as scikit-learn package[3]. We also tested our Cython implementation against a parallel Python (with *Numpy+Scipy+multiprocessing* packages) implementation, monitoring its scalability across different dataset sizes and number of available threads (ref. supp. Fig. 2). Equal to dataset sizes, the provided DNetPRO implementation is more than 10^4 faster than the Python one, allowing the processing of datasets with higher number of variables.supp. Fig. 2 shows sub-optimal results in terms of scalability, probably due to a non-optimal scheduling in the parallel section: the jobs are not equally distributed across the available threads, penalizing the code efficiency, and creating a bottleneck related to the slowest thread. We perform all the simulations considering a number of features equal to 90 and, thus, the parallel section distributes the 8100 ($N \times N$) iterations across the available threads: when the number of iterations is proportional to the number of threads used (12, 20 and 30 in our case), we have a maximization of the time performances.

The discussed pipeline and the entire set of codes developed in this work are publicly available at DNetPRO-pipeline Github repository.

Ranked pairs analysis and signature characterization

The analysis of variable pairs can easily lead to very large number of combinations (e.g., 10^8 pairs with gene expression microarrays containing about 2×10^4 probes). In this configuration, multiple pairs could achieve the same performance score (ref. supp. Fig. 3a): the classification metrics have the same cardinality as integer numbers, corresponding to the number of available samples (10^2–10^3 in many omics cases). Therefore, the ranking according to performances is characterized by multiple "plateaus", and the selection of variable pairs, based on a hard thresholding procedure, is highly influenced by this behavior. As in other cases of ranked values[4], we can fit these ranking distributions with a combination of power-law functions (ref. supp. Fig. 3b).

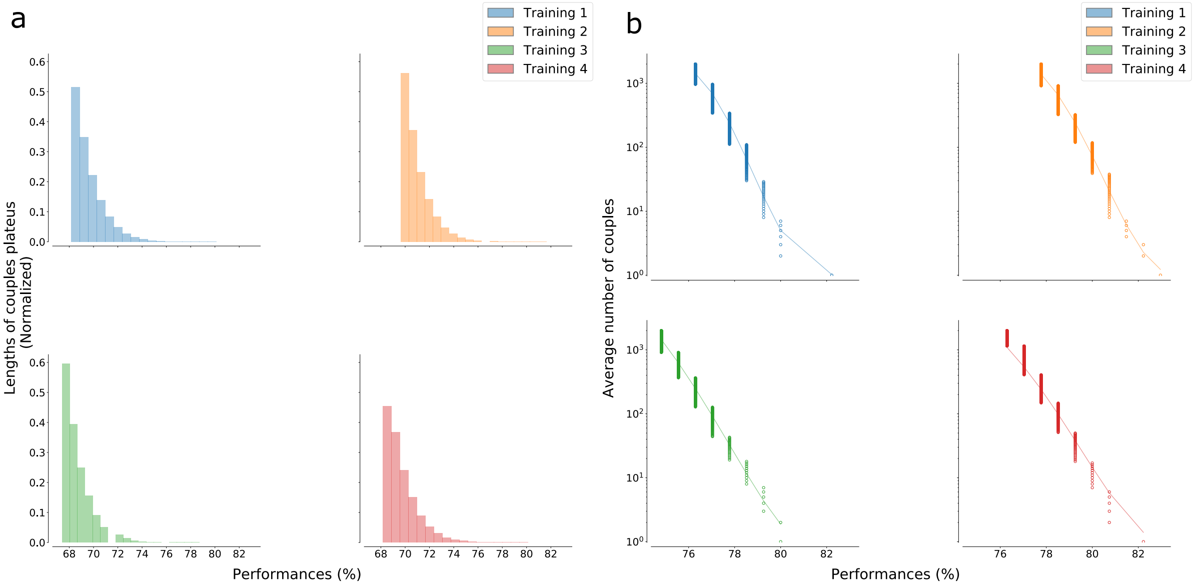

Supp. Fig. 3. Analysis of ranked pairs distributions according to the performance score obtained in the training step. (**a**) The distribution of plateau lengths is approximately exponential. (**b**) Average number of pairs with the same score value: this behavior is typical in ranking distribution and it can be fitted by the relation $f(x) = A(M+1-r)^b/r^a$ as shown in[4], where r is the rank value, M its maximum value, A a normalization constant and (a, b) two fitting exponents.

We observed that *star*-networks frequently appear in signatures generated by this procedure, with one center variable highly connected to the others (pendant nodes). This happens when a variable has a strong discriminating power, to which other, possibly less relevant, variables get linked due to noise fluctuations.

As stated in our work, we suggest that these variables (pendant nodes in the *star*-network) can be removed from the signature without affecting significantly its performance. The procedure can be applied for one single step (removing pendant nodes from the star configuration) or it can be applied recursively, until the signature becomes constituted only by the 2-core networks (i.e., with all nodes having degree ≥ 2).

Empirical analysis performed on real data has shown that the removal of these variables does not affect significantly the signature performance, allowing a significant reduction of its dimension. Since there is no clear theoretical explanation of this behavior, we suggest introducing this step only optionally in the DNetPRO workflow, since it is not easy to quantify the risk of losing relevant information from the removed variables.

The underlying idea is that the more connected the nodes are, the more the variables in the signature "work well" together, a plausible hypothesis given the linear sample separation surface provided by the Discriminant classifier. Moreover, the network structure of the signature suggests that additional measurements can be used for estimating the relevance of a variable as a function of its role in the network (e.g., node centrality such as degree or betweenness centrality) and therefore would be additional options for signature dimensional reduction (e.g., considering only the most central nodes). This approach has no theoretical foundation, and it has not been tested extensively in our study, even if some positive results have been found[5].

Description of the Synapse dataset

TCGA (The Cancer Genome Atlas) core sets of data used are available at the Synapse homepage (accession number syn300013, doi:10.7303/syn300013) created by Yuan et al.[6], and are composed of four tumor datasets: kidney renal clear cell carcinoma (KIRC), glioblastoma multiforme (GBM), ovarian serous cystadenocarcinoma (OV) and lung squamous cell carcinoma (LUSC).

The summary description of the datasets used in this work is reported in supp. Tab. 1.

Characterization of signatures overlap

In our applications, we divided the datasets into a training-test subdivisions and the signatures were extracted along a 10-fold cross-validation over the training sets. This kind of setup could, in the worst case, extract up to 10 totally different signatures (one for each split).

Cancer	mRNA	miRNA	Protein	Number of samples
GBM	AgilentG4502A	H-miRNA_8x15k	RPPA	
	17 814	533	[a]	210
KIRC	HiseV2	GA+Hiseq	RPPA	
	20 530	1 045	166	243
OV	AgilentG4502A	H-miRNA_8x15k	RPPA	
	17 814	798	165	379
LUSC	HiseqV2	GA+Hiseq	RPPA	
	20 530	1 045	174	121

Supp. Tab. 1. Description of the benchmark dataset used in the paper. The type of cancer, the platform with the number of probes and the number of samples per tumor type are shown.
[a] Missing data.

Starting from this large number of signatures, we evaluated the robustness of the DNetPRO algorithm in the feature identification, studying the overlap between them. From a statistical point-of-view, it is quite unlikely that the same set of features would be included into all the extracted signatures, especially on this application in which features represent gene expressions. On the other hand, the overlap of these signatures could highlight a statistical significance of some features, and thus genes related to the associated tumor.

For each fold we evaluated the average dimension of the signatures, and we computed the distribution of these dimensions for each cancer type. The global distributions of signature dimensions extracted by the DNetPRO algorithm on the four cancer types of the Synapse dataset are shown in supp. Fig. 4.

In supp. Fig. 5 the gene distributions obtained by the three methods (DNetPRO, K-best, and null models) are shown.

We performed a deeper analysis on the signatures of KIRC, looking for the overlaps between them. We analyzed the full list of genes included into the signatures, counting how many times the same gene appears. The total number of genes included into the signatures list is a subset (2 790) of the full list of probes in the dataset (20530). Despite the discrepancy between the signature dimensions, we had a core of 111 genes included in at least the 80% of the signatures. Looking for a more strict condition, we found a core of 74 genes in at least the 90% of the signatures and 20 of them are common in the 100%. For completeness, we mapped the list of 74 genes in the KEGG database to extract the associated pathways known in literature (ref. supp. Tab. 3). The list of genes also present in the KEGG database (65/74) is reported in supp. Tab. 2,in which we highlight the 15 out of 20 genes included in the 100% of the signatures with a †. We would remark that the overlap of the extracted signatures has a pure statistical meaning, since in our work we did not include any prior clinical/biological information in the algorithm, but it is useful to test the robustness of our method in identifying the same information across different simulations.

The low number of genes identified in the signatures allow us to map them on tumors-genes databases like the TISIDB[7]. The TISIDB stores all the known links between genes and tumors interactions using literature mining and high-throughput data analysis. We used the online version of the TISIDB, in which for each tumor type are reported the log-rank test values between the overall survival across human cancers and genes. We filtered out the genes which do not appear in the top-4 ranking list. We mapped only the list of 65 genes common to the full set of signatures, since they are the most statistical significant of our analysis. We found that a significant percentage (48/65, 74%) of the genes extracted by the DNetPRO signatures and common in at least the 90% of them, is already known in relation with the KIRC tumor by the TISIDB. The list of genes found in relation to the KIRC tumor is reported with the (*) in supp. Tab. 2.

References

1. Koster, J. & Rahmann, S. Snakemake - a scalable bioinformatics workflow engine. *Bioinformatics* **28**, 2520–2522 (2012).
2. Behnel, S. *et al.* Cython: The best of both worlds. *Computing in Science Engineering* **13**, 31 –39 (2011).
3. Pedregosa, F. *et al.* Scikit-learn: Machine learning in Python. *Journal of Machine Learning Research* **12**, 2825–2830 (2011).
4. Martínez-Mekler, G. *et al.* Universality of rank-ordering distributions in the arts and sciences. *PLOS ONE* **4**, 1–7 (2009). URL https://doi.org/10.1371/journal.pone.0004791.
5. Malvisi, M. *et al.* Combinatorial discriminant analysis applied to rnaseq data reveals a set of 10 transcripts as signatures of exposure of cattle to mycobacterium avium subsp. paratuberculosis. *Animals (Basel)* **10** (2020). URL https://pubmed.ncbi.nlm.nih.gov/32033399.

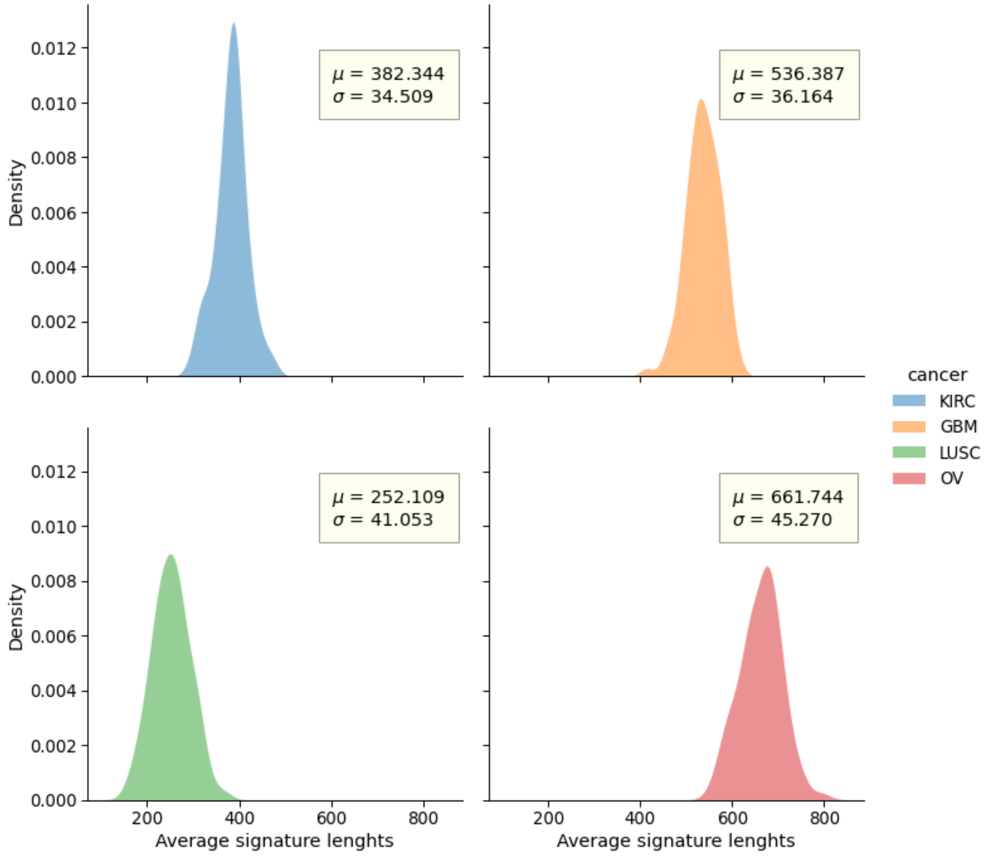

Supp. Fig. 4. Distribution of the signature dimensions extracted by the DNetPRO algorithm among the 100 simulations. For each simulation a total of 10 signatures were extracted and the average dimension was computed. For each cancer type (KIRC, GBM, LUSC, OV) we highlight the mean and standard deviation of the distribution.

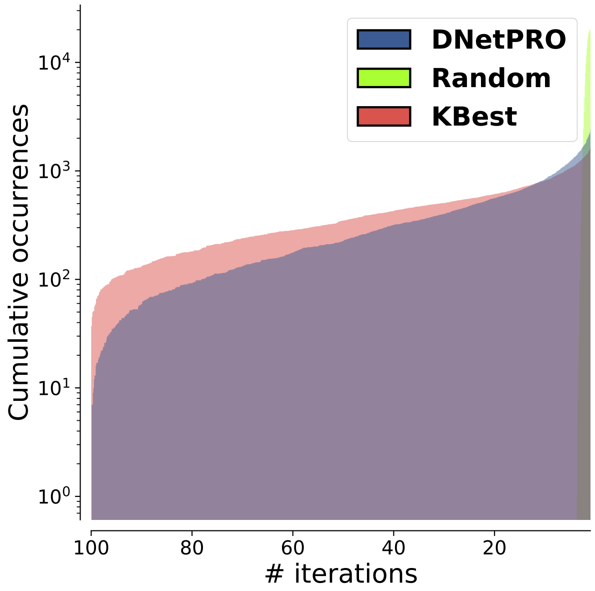

Supp. Fig. 5. Signatures overlap obtained in the KIRC mRNA datasets. Gene occurrences of the 1 000 DNetPRO signatures extracted from the Synapse pipeline (blue). Gene occurrences of the 1 000 K-best variables extracted from the Synapse pipeline (red): the number of genes (K) is the same of the corresponding DNetPRO signature. Gene occurrences of 1 000 random signatures (yellow).

Gene Symbol	Gene ID	Official Full Name
TROAP*†	hsa:10024	trophinin associated protein
CDK4*	hsa:1019	cyclin dependent kinase 4
SLC25A29*	hsa:123096	solute carrier family 25 member 29
LTB4R*†	hsa:1241	leukotriene B4 receptor
ANKLE1*	hsa:126549	ankyrin repeat and LEM domain containing 1
ATXN7L2*	hsa:127002	ataxin 7 like 2
UROC1	hsa:131669	urocanate hydratase 1
HRNBP3*	hsa:146713	RNA binding fox-1 homolog 3
EME1*	hsa:146956	essential meiotic structure-specific endonucle...
NCRNA00085	hsa:147650	sperm acrosome associated 6
RTKN2*	hsa:219790	rhotekin 2
SKA1*	hsa:220134	spindle and kinetochore associated complex sub...
GOLGA8A*	hsa:23015	golgin A8 family member A
GABBR1*	hsa:2550	gamma-aminobutyric acid type B receptor subunit 1
MYADML2	hsa:255275	myeloid associated differentiation marker like 2
BACE2*†	hsa:25825	beta-secretase 2
TAS2R20*	hsa:259295	taste 2 receptor member 20
WSB1*	hsa:26118	WD repeat and SOCS box containing 1
KAT2A*†	hsa:2648	lysine acetyltransferase 2A
KIF4B	hsa:285643	kinesin family member 4B
KRT73	hsa:319101	keratin 73
HOXC4	hsa:3221	homeobox C4
HOXC5*	hsa:3222	homeobox C5
BIRC5*	hsa:332	baculoviral IAP repeat containing 5
C8ORFK29†	hsa:340393	transmembrane protein 249
SLC16A12*	hsa:387700	solute carrier family 16 member 12
RNF207*	hsa:388591	ring finger protein 207
C1orf53*†	hsa:388722	chromosome 1 open reading frame 53
OR52D1	hsa:390066	olfactory receptor family 52 subfamily D member 1
ASNS*†	hsa:440	asparagine synthetase (glutamine-hydrolyzing)
NPPA	hsa:4878	natriuretic peptide A
GOLT1B*	hsa:51026	golgi transport 1B
ANAPC5*†	hsa:51433	anaphase promoting complex subunit 5
PTX3*	hsa:5309	paired like homeodomain 3
NUDT11*	hsa:55190	nudix hydrolase 11
HJURP*	hsa:55355	Holliday junction recognition protein
PRH1*	hsa:5554	proline rich protein HaeIII subfamily 1
ZNF692*	hsa:55657	zinc finger protein 692
LTB4R2	hsa:56413	leukotriene B4 receptor 2
PSMD2	hsa:5708	proteasome 26S subunit, non-ATPase 2
RARRES2*	hsa:5919	retinoic acid receptor responder 2
CHTF18*	hsa:63922	chromosome transmission fidelity factor 18
SGCB*†	hsa:6443	sarcoglycan beta
METT11D1	hsa:64745	methyltransferase like 17
THBS3*	hsa:7059	thrombospondin 3
ZNF26*†	hsa:7574	zinc finger protein 26
PABPC1L*†	hsa:80336	poly(A) binding protein cytoplasmic 1 like
KIAA1683	hsa:80726	IQ motif containing N
FGF23†	hsa:8074	fibroblast growth factor 23
CCNL2*†	hsa:81669	cyclin L2
CDCA3*	hsa:83461	cell division cycle associated 3
NUF2*	hsa:83540	NUF2 component of NDC80 kinetochore complex
ATAD3B*†	hsa:83858	ATPase family AAA domain containing 3B
MYCBPAP	hsa:84073	MYCBP associated protein
PPFIA4*	hsa:8497	PTPRF interacting protein alpha 4
NPFF*	hsa:8620	neuropeptide FF-amide peptide precursor

Gene Symbol	Gene ID	Official Full Name
STX16*	hsa:8675	syntaxin 16
CCNA2*	hsa:890	cyclin A2
GYG2*†	hsa:8908	glycogenin 2
TIMELESS*	hsa:8914	timeless circadian regulator
CCNF*	hsa:899	cyclin F
LASS5	hsa:91012	ceramide synthase 5
EXO1	hsa:9156	exonuclease 1
NUMBL*	hsa:9253	NUMB like endocytic adaptor protein
KIF23*	hsa:9493	kinesin family member 23

Supp. Tab. 2. List of the genes included in at least the 90% of the signatures extracted by the DNetPRO algorithm along the 100 simulations on the KIRC dataset. The full list of 74 genes was mapped on the KEGG database to extract the gene information and only the successful matches (65/74) is reported in the table. We mark with † the genes included in the 100% of the DNetPRO signatures. We mark with (*) the genes for which we found a correspondence in the TISIDB with KIRC tumor (in the top-4 ranking). Only SGCB and SLC16A12 are associated with long term of survival, while all the other genes are associated to short terms.

KEGG Pathway	Pathway	Occurrences
hsa:05166	Human T-cell leukemia virus 1 infection	4
hsa:05200	Pathways in cancer	4
hsa:04080	Neuroactive ligand-receptor interaction	4
hsa:04110	Cell cycle	3
hsa:04151	PI3K-Akt signaling pathway	3
hsa:05165	Human papillomavirus infection	3
hsa:05169	Epstein-Barr virus infection	3
hsa:05203	Viral carcinogenesis	3
hsa:01100	Metabolic pathways	3

Supp. Tab. 3. List of pathways associated to the genes reported in supp. Tab.2. For each pathway we report the number of occurrences along the gene list. The pathways are sorted by the occurrence values.

6. Yuan, Y. *et al.* Assessing the clinical utility of cancer genomic and proteomic data across tumor types. *Nature Biotechnology* **32**, 644–652 (2014). URL https://www.nature.com/articles/nbt.2940.

7. Ru, B. *et al.* TISIDB: an integrated repository portal for tumor–immune system interactions. *Bioinformatics* (2019). URL https://doi.org/10.1093/bioinformatics/btz210. Btz210, http://oup.prod.sis.lan/bioinformatics/advance-article-pdf/doi/10.1093/bioinformatics/btz210/28492245/btz210.pdf.
